# Prostate enlargement and altered urinary function are part of the aging process

**DOI:** 10.18632/aging.101938

**Published:** 2019-05-13

**Authors:** Teresa T. Liu, Samuel Thomas, Dalton T. Mclean, Alejandro Roldan-Alzate, Diego Hernando, Emily A. Ricke, William A. Ricke

**Affiliations:** 1 Department of Urology, University of Wisconsin – Madison, Madison, WI 53705, USA; 2 K12 Kure, University of Wisconsin – Madison, Madison, WI 53706, USA; 3 Molecular and Environmental Toxicology, University of Wisconsin – Madison, Madison, WI 53706, USA; 4 Cancer Biology, University of Wisconsin – Madison, Madison, WI 53706, USA; 5 Department of Mechanical Engineering, University of Wisconsin – Madison, Madison, WI 53706, USA; 6 Department of Radiology, University of Wisconsin – Madison, Madison, WI 53705, USA; 7 Department of Medical Physics, University of Wisconsin – Madison, Madison, WI 53705, USA; 8 George M. O’Brien Center of Research Excellence, University of Wisconsin – Madison, Madison, WI 53705, USA

**Keywords:** benign prostatic hyperplasia, mouse models, lower urinary tract dysfunction, ultrasound, magnetic resonance imaging

## Abstract

Prostate disease incidence, both benign and malignant, directly correlates with age. Men under 40 years of age are rarely diagnosed with benign or malignant prostate disease, while 90% of men over the age of 80 have histological evidence of benign disease (benign prostatic hyperplasia; BPH). Although rodent models have been invaluable in the study of disease progression and treatment efficacy, the effect of age is often not considered. In examining aged (24-month-old) mice, we observed changes within the lower urinary tract that is typically associated with lower urinary tract dysfunction (LUTD) similar to models of BPH. In this study, we identify LUTD using functional testing as well as various imaging technologies. We also characterize the histological differences within the lower urinary tract between young (2-month-old) and aged mice including proliferation, stromal remodeling, and collagen deposition. Additionally, we examined serum steroid hormone levels, as steroid changes drive LUTD in mice and are known to change with age. We conclude that, with age, changes in prostate function, consistent with LUTD, are a consequence. Therapeutic targeting of endocrine and prostatic factors including smooth muscle function, prostate growth and fibrosis are likely to reestablish normal urinary function.

## Introduction

The prostate is a male accessory sex gland that has a high incidence of disease, both benign and malignant, coincident with aging. Prostate disease is so common it is anecdotally considered a part of the aging process [1–3]. Prostate cancer is the second leading cause of cancer death in American men and is rarely diagnosed prior to the age of 40 [4]. Benign prostatic hyperplasia (BPH) is a disease that develops in men as they age; nearly 90% of men over 80 years of age are living with this condition [3,5]. Although a non-malignant disease, many men with BPH develop lower urinary tract symptoms (LUTS), including urgency, frequency, and nocturia, that significantly decrease their quality of life. The cost of treatment for BPH in the United States is more than $4 billion annually [6–8]. The cause of LUTS in BPH can be multifactorial. While most commonly attributed to an increase in stromal and/or epithelial proliferation leading to the formation of nodules that impinge on the urethra, changes in smooth muscle contractility and an increase in fibrosis have also been implicated in BPH/LUTS. Additionally, while the prostate is generally considered an androgen-regulated organ, both androgens and estrogens are critical in the maintenance of the normal prostate. Changes within the prostate’s hormone milieu have been implicated in disease initiation and progression. As men age, circulating levels of testosterone (T) decrease while levels of estrogen (E) remain constant or increase, leading to a lowered T to E (T:E) ratio [9–11]. Several studies have examined the changes of serum sex steroid hormones through aging and how these changes are inversely related to prostate disease [12–14].

Mouse models have been invaluable in the study of a variety of human diseases. We have developed a hormone-induced mouse model that recapitulates the aging hormone environment in men [15,16]. The compressed T and 17β-estradiol (E2) pellets release hormones at a rate in which the concentration of T decreases over time while E2 remains constant. This model is used to demonstrate lower urinary tract dysfunction (LUTD) similar to that of human BPH disease as well as hormonal carcinogenesis and progression when used in conjunction with renal capsule xenografts [15,17]. The mice in these models demonstrate an increase in prostate and bladder mass, prostate and bladder volume, and an increase in collagen remodeling [17]. Previous studies using this aging model have evaluated changes within mice between 2-6 months in age, corresponding to the human equivalent of 20-30 years [18]. Since BPH is a disease that predominantly affects men over the age of 55, using mice in that correlative age range (18-24-month-old mice) could give better insight into disease progression. Additionally, cellular changes within the prostate have been examined in the context of aging, but these changes were not associated with urinary function [19].

In this study, we examine the changes within the lower urinary tract that occur under normal aging conditions in mice. Although the mouse prostate and human prostates differ in anatomy, we have previously identified regions within the mouse lower urinary tract that are similar to the human prostate anatomy [17,20]. By identifying baseline changes in proliferation, extracellular matrix deposition, and steroid hormones and receptors, we can better understand the contribution of age to disease phenotype.

## RESULTS

### Characterization of the lower urinary tract in aging mice

**Figure 1:**
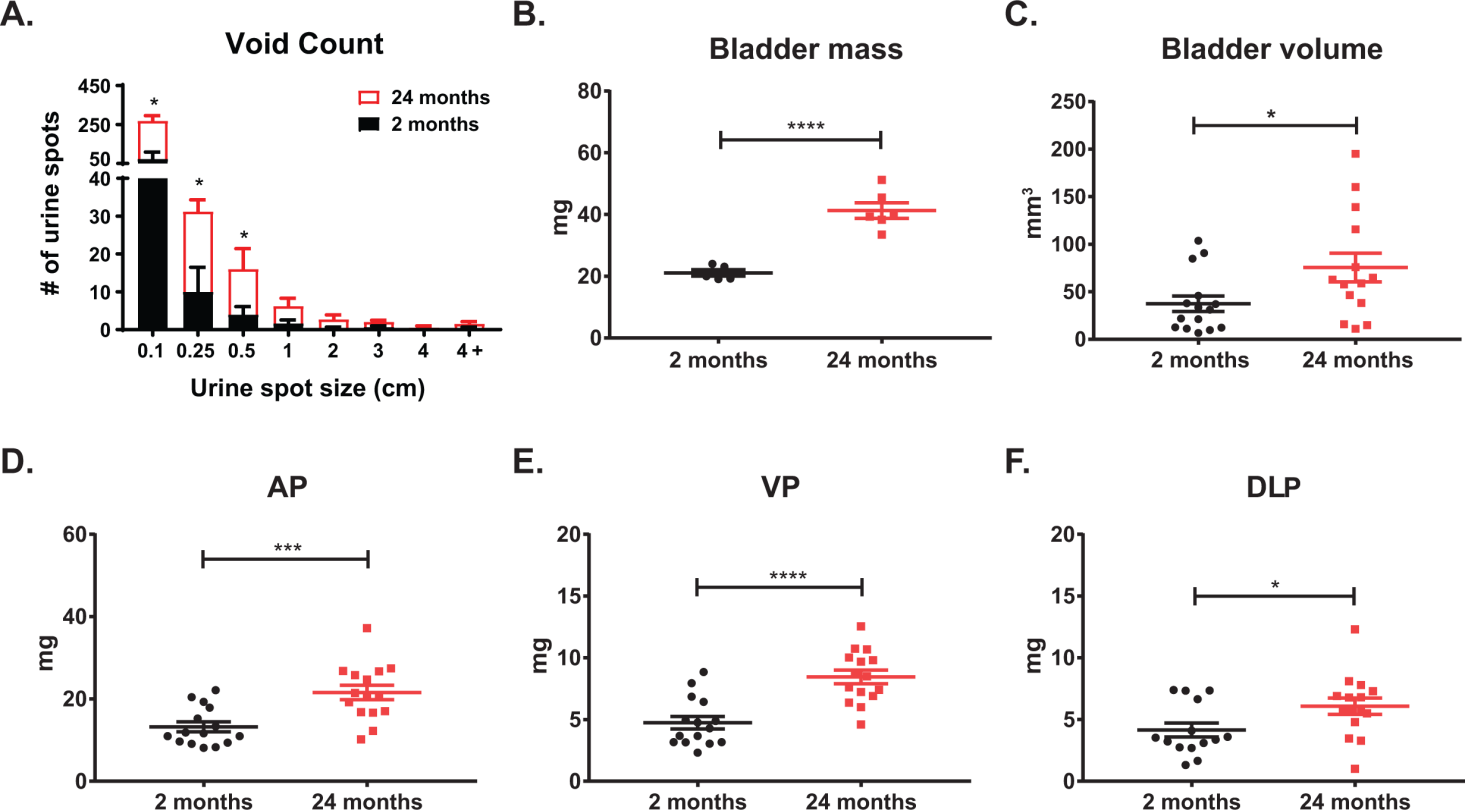
**Lower urinary tract measurements show age related changes.** (**A**) Aging mice show a significant increase in total urine spots as well as an increase in smaller urine spots as measured by void spot assay. (**B**) Aging significantly increases bladder mass. (**C**) Aging significantly increases bladder volume as calculated from caliper measurements. (**D**) Age significantly increases anterior prostate (AP) mass. (**E**) Aging significantly increases ventral prostate (VP) mass. (**F**) Aging significantly increases dorsolateral prostate (DLP) mass. *, p-value < 0.05; ***, p-value < 0.001; ****, p-value < 0.0001.

The lifespan of different strains of mice have been extensively characterized [21]. Generally, the period between three to six months in mice corresponds to humans at approximately 20-30 years of age. In C57Bl6/J mice, 75% survive past 2 years of age, and only 50% survive past 27 months; mice between 18-24 months of age correspond to humans 56-69 years of age [22]. Since wild-type mice less than a year old do not typically develop prostatic diseases (benign or malignant), a comprehensive analysis of the physiology and histology of the lower urinary tract with aging has not been rigorously assessed. Using void spot assays (VSA) to assess urinary function in wild-type, untreated mice at 2 months (young) and 24 months (aged) of age, we observed a significant increase in the number of urine spots in the aged cohort of mice compared to the young (Figure 1). Additionally, the aged cohort has a significant number of smaller urine spots compared to the young cohort consistent with LUTD. Upon examination of the dissected lower urinary tracts, we observed a significant increase in bladder mass and retained urine volume (Figure 1B-C). Examination of the separate prostate lobes [anterior (AP), ventral (VP), dorsolateral (DLP)] showed a significant increase in mass in the aged cohort compared to the young cohort (Figure 1D-F). These results are similar to changes we found previously with the hormone induced mouse model of LUTD [17]. When these morphometric measurements were normalized to body mass, bladder mass, total prostate lobes (hemiprostate), AP, and VP remained statistically significant with aging (Table 1). This suggests that normal aging recapitulates symptoms of BPH with corresponding changes in prostate and bladder, independent of body mass.

**Table 1:** Morphometric measurements of mouse bladder and prostate normalized to body mass.

**Measurement**	**Young (mg)**	**Aged (mg)**	**p-value**
Body mass (BM)	27.54 ± 0.61	34.66 ± 1.04	**< 0.0001**
Bladder mass	0.70 ± 0.04	1.10 ± 0.09	**0.0037**
Bladder volume	1.20 ± 0.26	2.25 ± 0.44	0.0512
Hemiprostate	0.81 ± 0.07	1.05 ± 0.61	**0.0136**
AP	0.47 ± 0.36	0.63 ± 0.05	**0.0207**
VP	0.17 ± 0.02	0.25 ± 0.02	**0.0044**
DLP	0.17 ± 0.03	0.18 ± 0.02	0.7807

The body mass of aged mice was significantly different from young mice, so the bladder and prostate measurements were normalized against body mass. Masses are reported as mean ± standard error, and statistically significant p-values are bolded.

### Aging leads to changes in urethra lumen and flow velocity

**Figure 2:**
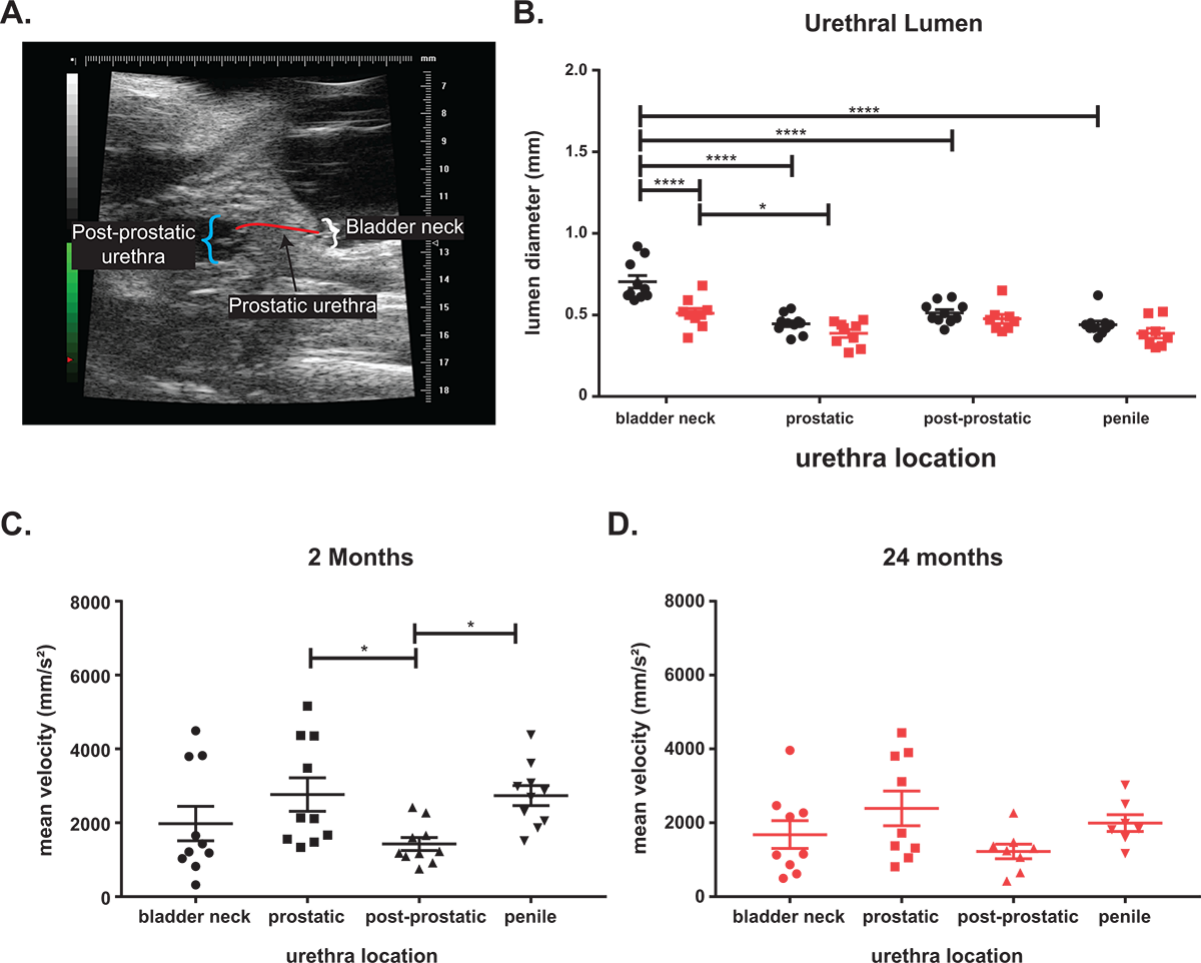
**Aging leads to voiding changes as measured by ultrasound.** (**A**) Ultrasound image showing the three urethra regions. (**B**) The urethral diameter is the largest at the bladder neck with a significant drop at the prostatic urethra before the diameter increases at the post-prostatic urethra in young mice. The post-prostatic urethra, while larger than the prostatic urethra is still significantly smaller than the bladder neck diameter. The penile urethra is slightly smaller than the post-prostatic urethra but significantly smaller than the bladder neck. There is a significant decrease in bladder neck diameter with age and a slight decrease at all other locations with age. The decrease of lumen diameter between bladder neck and diameter is still significant in aged mice. (**C**) The mean velocity through the urethra of young mice show a significant decrease between prostatic and post-prostatic and a significant increase between post-prostatic and penile consistent with a narrowing of lumen diameter. (**D**) The mean velocity is highest at the prostatic urethra in aged mice, but the changes in velocity is lost with age. The mean velocity of flow in aged mice is lower than mean velocity in young mice. *, p-value < 0.05; ***, p-value < 0.001; ****, p-value < 0.0001.

One potential cause of LUTD is bladder outlet obstruction (BOO; i.e. a narrowing of the urethral lumen), leading to changes in urinary flow and pressure that further remodels (i.e. hypertrophy) the bladder. Using ultrasound, we measured the lumen diameters and flow velocity throughout the urinary tract [23]. We separated the urethra into four discrete regions: bladder neck (pre-prostatic), prostatic, post-prostatic, and penile urethras (Figure 2A). The bladder neck is the triangular (i.e. funnel) region where the bladder connects to the urethra. The prostatic urethra begins with the formation of the rhabdosphincter around the urethra and ends with the opening of the joining of the ejaculatory ducts into the lumen [17,20,24]. The post-prostatic urethra which follows the prostatic urethra, has the largest lumen diameter, and ends at narrowing into the penile urethra. The penile urethra begins at the end of the post-prostatic urethra and continues through the sigmoid flexure and penis [25]. The urethral lumen diameter is the largest at the bladder neck and significantly decreases when it transitions to the prostatic urethra (Figure 2B). The urethral lumen then increases significantly at the post-prostatic urethra. We found a significant decrease in the diameter of the bladder neck in aged mice compared to young. Examination of the mean velocity (the rate of flow through the urethra) in the young mice show a significantly higher velocity in the regions where the urethral lumen is smaller (Figure 2C). Upon aging, while there is no significant difference in lumen diameter, the velocity change between the prostatic and the post-prostatic urethra is no longer significant (Figure 2D). This change with age suggests the prostatic urethra as a potential chokepoint, leading to flow changes, could underlie the observed LUTD.

### Aging leads to changes in urethral length

**Figure 3:**
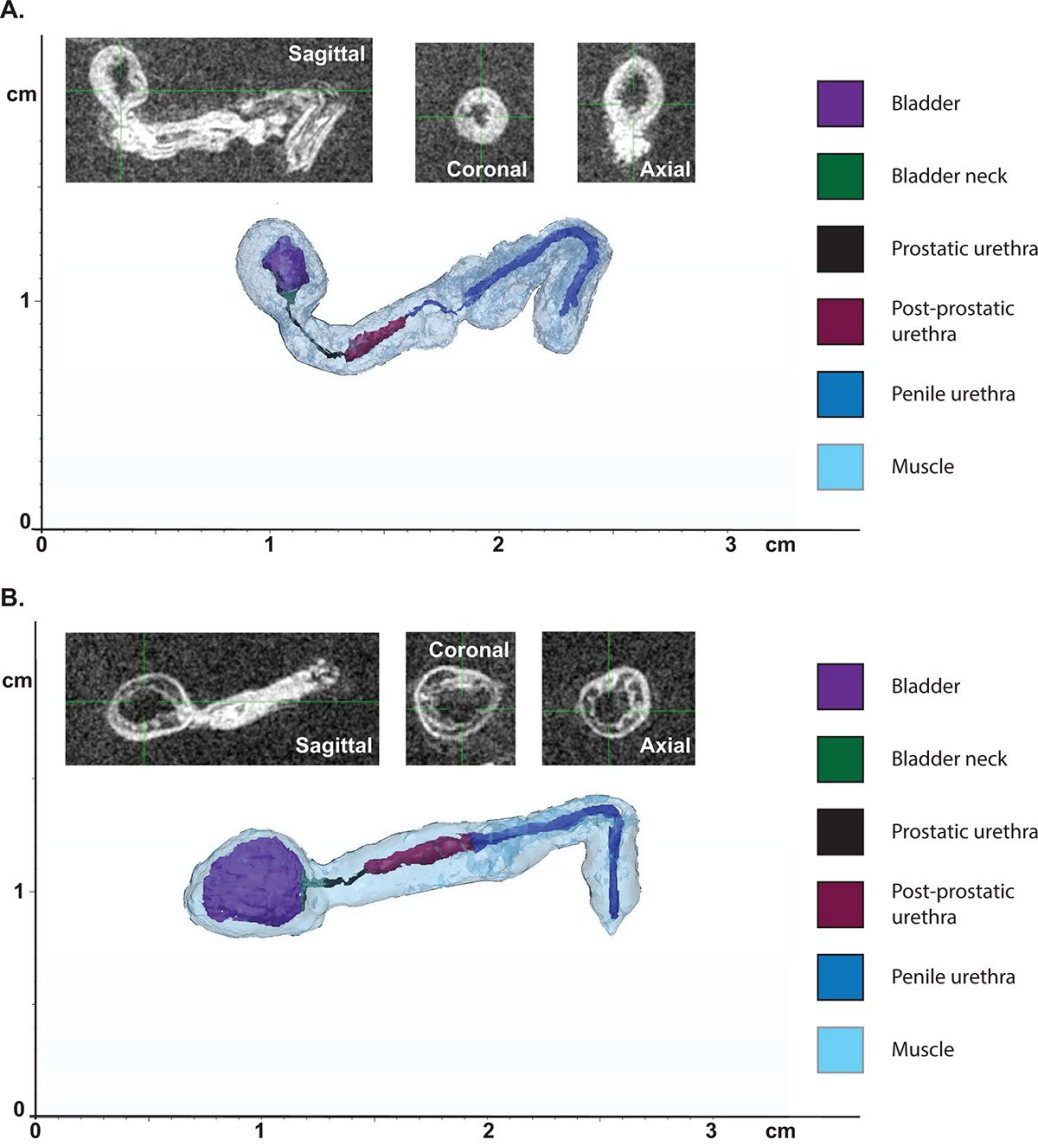
**Aging leads to changes in urethral shape and area as measured by MRI.** (**A**) Representative 3D reconstruction of the bladder and urethra from a young mouse. (**B**) Representative 3D reconstruction of the bladder and urethra from an aged mouse. Insets show MRI images in three planes used for the reconstruction.

Previously, in order to examine overall changes in urethral lumen diameter within the mouse, histological sections at 5µm had to be stained, imaged, and structures hand-traced for 3D reconstruction [26,27]. Magnetic resonance imaging (MRI) allows for high resolution imaging of *ex vivo* tissues and visualization in 3D which allows for more precise measurements. The lower urinary tracts of young and aged mice were subjected to MRI, and 3D reconstruction of the tract performed using MIMICS software (Figure 3A-B). The lumen diameter in the prostatic urethra was unchanged in aged mice compared to young mice. However, there was a significant increase in lumen length in response to age (Table 2). There were also notable changes in the total volume of the prostatic urethra. Examination of the effect of age on bladder volume showed a significant increase in overall volume, detrusor volume, as well as lumen volume. This suggests that the increase in bladder volume not only corresponds to the hypertrophy of the bladder wall, but there is also an increase in retained urine volume within the bladder.

**Table 2:** Measurements taken from MRI images.

**Measurement**	**Young**	**Aged**	**p-value**
Bladder volume	44.19 ± 7.07	89.11 ± 5.75	**0.0021**
Detrusor volume	33.38 ± 2.67	57.42 ± 3.12	**< 0.001**
Bladder lumen volume	10.81 ± 5.56	31.70 ± 3.19	**0.02**
Bladder neck length	1.19 ± 0.16	0.99 ± 0.14	0.38
Bladder neck diameter	0.27 ± 0.04	0.48 ± 0.19	0.27
Bladder neck volume	0.19 ± 0.03	0.20 ± 0.08	0.83
Prostatic urethra length	1.31 ± 0.15	2.24 ± 0.24	**0.01**
Prostatic urethra diameter	0.3 ± 0.06	0.34 ± 0.49	0.62
Prostatic urethra volume	0.06 ± 0.02	0.12 ± 0.03	0.18
Post-prostatic urethra length	4.75 ± 0.46	5.13 ± 0.89	0.70
Post-prostatic urethra diameter	0.7 ± 0.04	0.90 ± 0.25	0.39
Post-prostatic urethra volume	1.80 ± 0.36	2.47 ± 1.60	0.66
Penile urethra length	11.70 ± 1.10	10.66 ± 2.11	0.65
Penile urethra diameter	0.43 ± 0.07	0.54 ± 0.07	0.32
Penile urethra volume	2.03 ± 0.91	2.31 ± 0.60	0.81
Total lumen volume	3.89 ± 1.18	4.90 ± 2.20	0.68

The 3D reconstructed lower urinary tracts were segmented and measured (mean ± standard error), and statistically significant p-values are bolded. Lengths and diameters are reported as mm; volumes are reported as mm^3^.

*Only pathways known to be important for oocyte quality were selected.

### The effect of aging on smooth muscle

**Figure 4:**
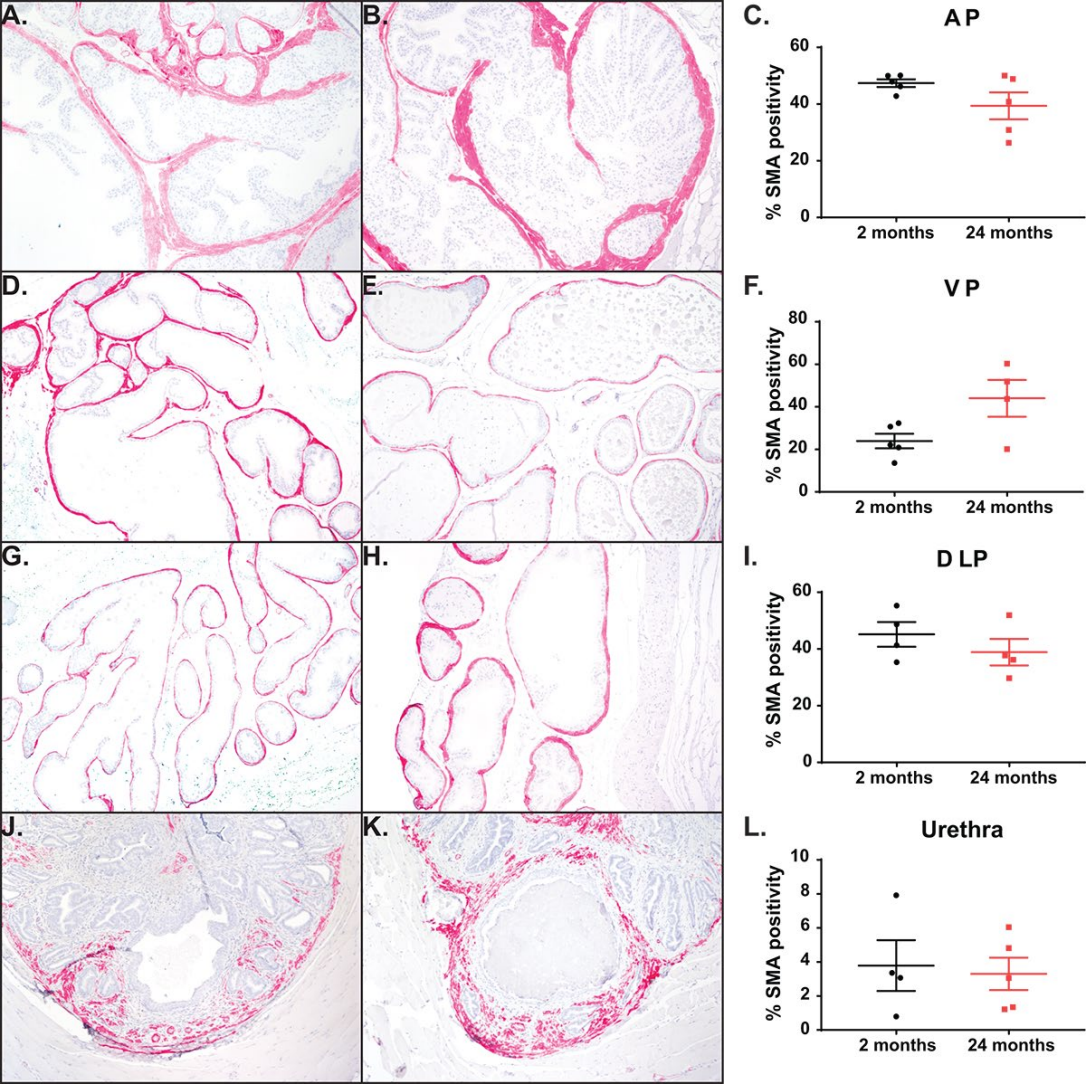
**Aging has no impact on percentage of smooth muscle cells.** (**A**) Representative image of smooth muscle α-actin (SMA) staining within the AP in young mice. (**B**) Representative image SMA staining within the AP in aged mice. (**C**) Quantitation of percent SMA within the AP shows no difference with age. (**D**) Representative image of SMA staining within the VP in young mice. (**E**) Representative image SMA staining within the VP in aged mice. (**F**) Quantitation of percent SMA within the VP shows no difference with age. (**G**) Representative image of SMA staining within the DLP in young mice. (**H**) Representative image SMA staining within the DLP in aged mice. (**I**) Quantitation of percent SMA within the DLP shows no difference with age. (**J**) Representative image of SMA staining within the prostatic urethra of young mice. (**K**) Representative image SMA staining within the prostatic urethra of aged mice. (**L**) Quantitation of percent SMA around the urethral lumen within the prostatic urethra shows no difference with age.

One effective treatment of BPH is the use of α-adrenergic blockers to relax the smooth muscle in the bladder and the prostate. To examine the effect of aging on the distribution of smooth muscle within the mouse, we quantified the percentage of smooth muscle α-actin (SMA) within the prostate lobes (Figure 4A-I). We observed that the smooth muscle found within the prostate stroma remains unchanged with age. While the anatomy of the mouse prostate (lobular) is different than that of the human (capsular), the prostatic urethra region has the smallest urethral luminal diameter and is encompassed by the rhabdosphincter similar to the encapsulated human prostate [28]. Examination of the smooth muscle immediately surrounding the prostatic urethral lumen shows no difference in percentage of SMA between the young and aged cohort (Figure 4J-L). This suggests that smooth muscle within the stroma is not contributing to the age related LUTD seen in mice.

### Prostate proliferation rate throughout aging

**Figure 5:**
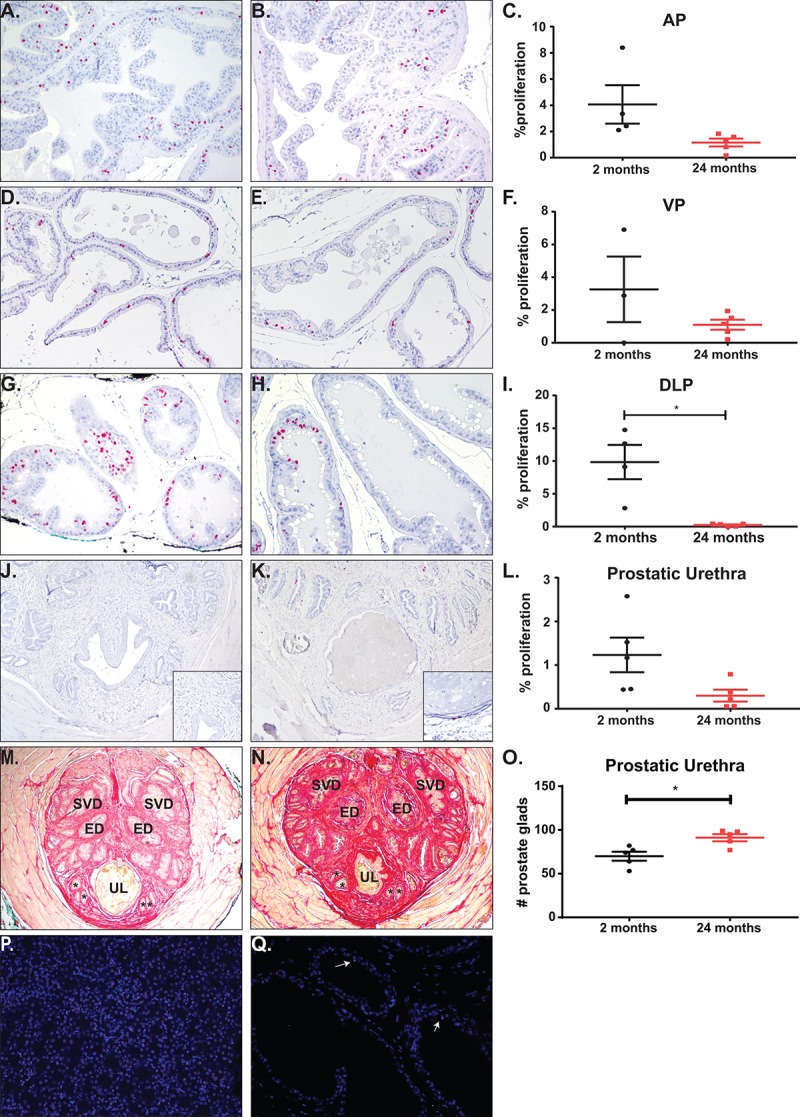
**Total proliferation increases with age with no change in proliferation or apoptosis rates.** (**A**) Representative image of BrdU accumulation within the AP of young mice. (**B**) Representative image of BrdU accumulation with the AP of aged mice. (**C**) Quantitation of percent proliferation within the AP shows no significant change in proliferation rate with age. (**D**) Representative image of BrdU accumulation within the VP of young mice. (**E**) Representative image of BrdU accumulation with the VP of aged mice. (**F**) Quantitation of percent proliferation within the VP shows no significant change in proliferation rate with age. (**G**) Representative image of BrdU accumulation within the DLP of young mice. (**H**) Representative image of BrdU accumulation with the DLP of aged mice. (**I**) Quantitation of percent proliferation within the DLP shows a significant decrease in proliferation rate with age. (**J**) Representative image of BrdU accumulation around the urethral lumen within the prostatic urethra of young mice. Inset (**K**) Representative image of BrdU accumulation around the urethral lumen within the prostatic urethra of aged mice. (**L**) Quantitation of percent proliferation around the urethral lumen within the prostatic urethra shows no significant change in proliferation rate with age. (**M**) Representative image of the prostatic urethra of young mice. * denote representative prostate glands within the rhabdosphincter. (**N**) Representative image of the prostatic urethra of young mice. *, prostate glands; ED, ejaculatory duct; SVD, seminal vesicle duct; UL, urethral lumen. (**O**) Quantitation of prostate glands within the prostatic urethra shows a significant increase in gland number with age. (**P**) Staining for apoptosis of a kidney section with induced DNA breaks shows a significant percentage of TUNEL positive cells (green). (**Q**) Representative image of apoptosis in prostate tissue shows little to no TUNEL positive cells (white arrows).

While the prostate is thought to be a quiescent organ post-puberty, we discovered the prostate mass increased significantly between the young and the aged mice (Figure 1D-F). Previous studies have shown that prostate proliferation in mature mice is ~1% [29,30]. To quantify the amount of proliferation that occurs within the mice during a four-week treatment, we treated mice orally with BrdU in water for a week and examined proliferation changes at the end of the study. Anterior and ventral prostates were undergoing ~1-5% proliferation, regardless of age (Figure 5A-F). Although not significantly different, there is a trend towards a decrease in proliferation with age. Dorsolateral prostates showed a significant increase in BrdU incorporation in the young mice as compared to the aged mice (Figure 5G-I). This higher proliferation rate in the young mice could indicate that DLP maturation to quiescence occurs after 2 months of age. We also examined proliferation of prostatic tissue immediately surrounding the urethra at the midpoint of the prostatic urethra. Previous studies have shown that an increase in prostate ducts within the region are associated with urinary dysfunction [17]. Examination of BrdU incorporation within the prostatic urethra shows no change in proliferation rate in the aged mouse cohort compared to the young mice (Figure 5J-L). However, examination of prostate ducts within the midpoint of the prostatic urethra showed a significant increase in duct number without a corresponding change in BrdU incorporation (Figure 5M-O). This suggests that while the rate of proliferation does not change with age within the prostate lobes and prostatic urethra, the constant proliferation over time does increase prostate ducts and may be contributing to LUTD. Examination of apoptosis within the prostate lobes and the midpoint of the prostatic urethra showed a < 0.5% apoptosis with no difference between young and aged mice (Figure 5P-Q).

### Aging leads to changes in the extracellular matrix

**Figure 6:**
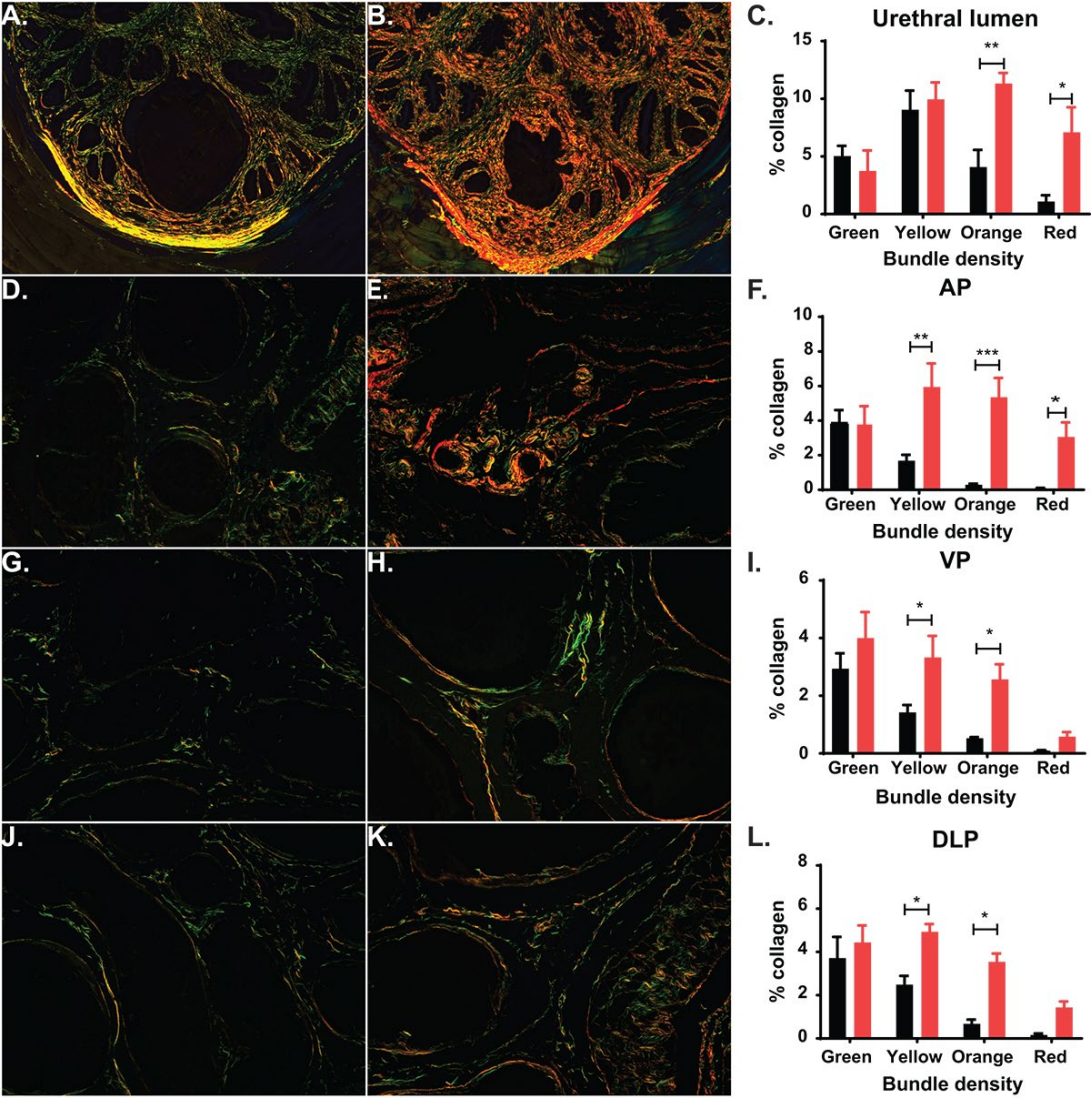
**Aging leads to changes in collagen deposition.** (**A**) Representative image of picrosirius red (PSR) staining of prostatic urethra specifically around the urethral lumen show predominantly green and yellow collagen fibers in young mice. (**B**) Representative image of PSR staining show predominantly orange and green collagen fibers in aged mice. (**C**) Quantitation of collagen fibers in the prostatic urethra show a statistical increase of orange and red collagen bundles in aged mice. (**D**) Representative image of PSR staining show predominantly green collagen fibers within the AP of young mice. (**E**) Representative image of PSR staining show predominantly yellow, orange, and red collagen bundles within the AP of aged mice. (**F**) Quantitation of collagen fibers within the AP shows a statistical increase in yellow, orange, and red collagen bundles and an overall increase in total collagen in aged mice. (**G**) Representative image of PSR staining show predominantly green collagen fibers within the VP of young mice. (**H**) Representative image of PSR staining show predominantly yellow and orange collagen bundles within the VP of aged mice. (**I**) Quantitation of collagen fibers within the VP shows a statistical increase in yellow and orange collagen bundles and an overall increase in total collagen in aged mice. (**J**) Representative image of PSR staining show predominantly green collagen fibers within the DLP of young mice. (**K**) Representative image of PSR staining show predominantly yellow and orange collagen bundles within the DLP of aged mice. (**L**) Quantitation of collagen fibers within the DLP shows a statistical increase in yellow and orange red collagen bundles and an overall increase in total collagen in aged mice. *, p-value < 0.05; **, p-value < 0.01

In human prostate, an increase in collagen deposition and fibrosis has been identified as a contributing factor of urinary dysfunction [31]. In the mouse, previous studies have shown a significant increase in fibrosis and collagen deposition within the prostatic urethra in mice experiencing LUTD, suggesting that collagen changes can alter normal urination [17,32,33]. We calculated collagen deposition at the midpoint of the prostatic urethra and in the prostate lobes by quantifying birefringence of picrosirius red (PSR) staining under circular polarized light. After exploring the periurethral region of the mouse prostatic urethra, we observed an age-related, significant increase in total collagen, with the most significant increases in the thickest collagen bundles (orange and red; Figure 6A-C). Additionally, examination of the prostate lobes showed a significant increase in collagen of the aged mice corresponding to the thicker collagen bundles (yellow, orange, and red; Figure 6D-L). This suggests that collagen is accumulated over time, leading to changes within the prostate lobes and the prostatic urethra. This could also contribute to the LUTD observed in the aged mice.

### Steroid hormone changes through aging

**Figure 7:**
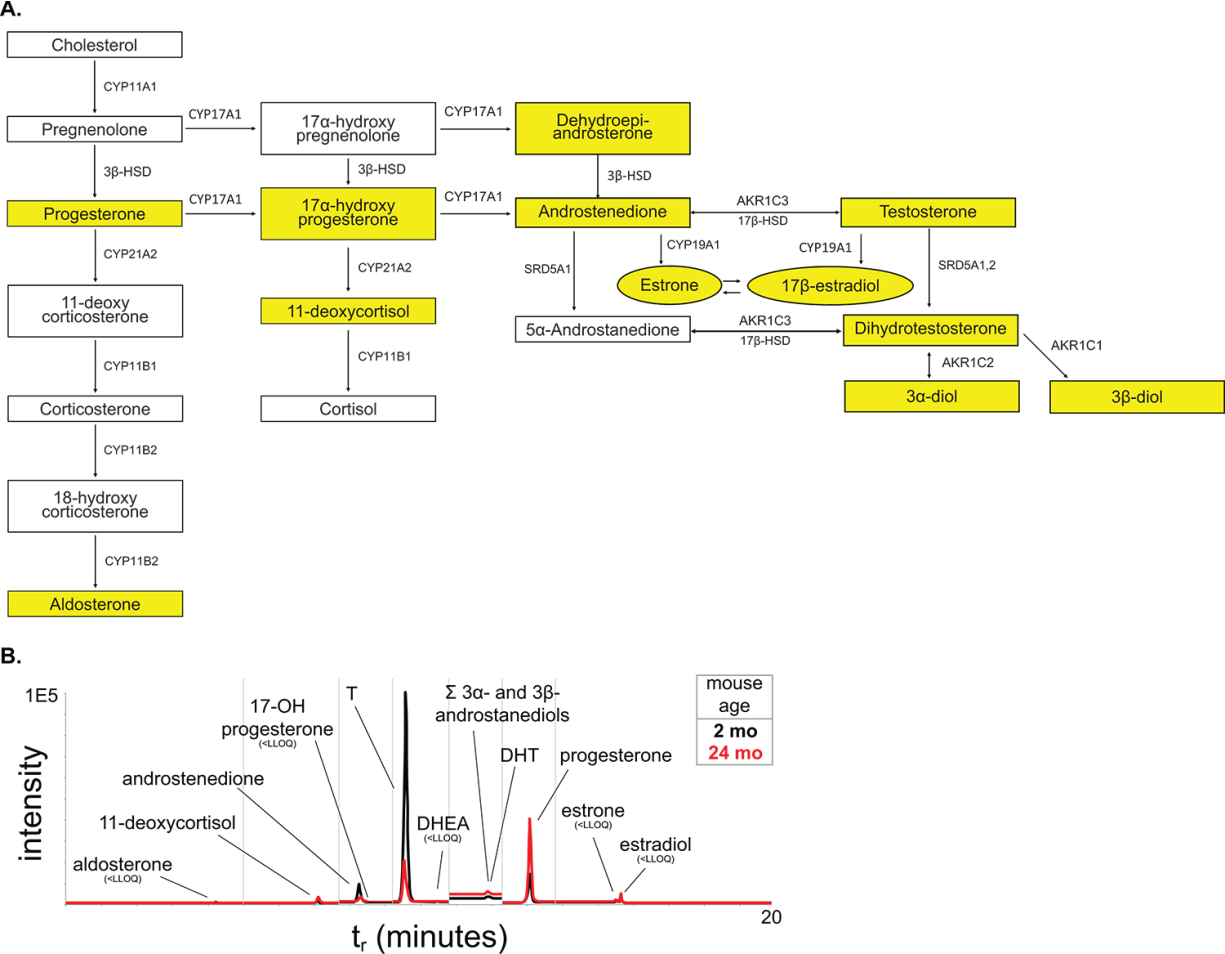
**Steroid hormone metabolism pathway.** (**A**) The steroid hormone pathway with the analytes represented in our LC-MS^2^ steroid hormone panel highlighted in yellow. (**B**) Representative chromatogram of the steroid panel analytes with young in black and aged in red.

One risk factor for prostate disease in humans is the changing hormone environment associated with aging [9]. Although the prostate is generally considered an androgen-dependent organ, estrogens and estrogen receptor signaling have been implicated in disease progression [34,35]. To examine the circulating steroid hormone environment, we applied a liquid chromatography – tandem mass spectrometry (LC-MS^2^) method for the quantification of a panel of twelve steroid hormones (Figure 7A). Previous studies in aging mice have shown that testosterone (T) levels decrease in mice through aging similar to humans as measured by radioimmunoassay and ELISA [11,15]. Additionally, the variability of serum T in mice decreased significantly with aging [36]. Our LC-MS^2^ studies show a 50% decrease in serum T through aging with a significant difference in variance (Figure 7B, Table 3). Additionally, examination of the downstream steroid hormone dihydrotestosterone (DHT) shows a similar downward trend with age. We were unable to detect circulating levels of estrogens at a lower limit of quantification (LLOQ) of ~0.6 pg/mL, indicating that circulating levels of estrogens in male C57Bl/6 mice are low. This suggests that the decrease of T corresponds to a decrease in AR activation. Additionally, the decrease in T, in conjunction with the changes in LUTD, suggests that mice do develop prostate disease very similar to human BPH.

**Table 3:** Circulating serum steroid hormone concentration from young and aged mice as measured by the LC-MS^2^ panel.

	Steroid concentration (ng/mL; x̄ ± SD)		LC-MS/MS analytical details				
Steroid analyte	young	aged	retention time (min)	MRM 1	MRM 2	LLOQ (ng/mL)	ULOQ (ng/mL)
11-deoxycortisol (11-DCSOL)	**0.040 ± 0.037**	**0.14 ± 0.11**	7.10	347.0-96.8	347.0-109.0	5.5E-03	2.3E+01
17-hydroxyprogesterone (17-OHP)	n/a	n/a	8.5	330.9-109.1	330.9-97.1	n/a	n/a
progesterone (PROG)	0.80 ± 0.39	1.3 ± 1.0	13.09	314.9-97.0	314.9-109.1	3.7E-03	1.9E+01
aldosterone (ALDO)	< 0.029	< 0.029	4.26	361.1-343.1	361.1-77.0	2.9E-02	2.3E+01
androstenedione (ANDRO)	0.10 ± 0.13	0.10 ± 0.09	8.27	287.1-97.2	287.1-109.1	3.7E-03	1.9E+01
dehydroepiandrosterone (DHEA)	< 0.029	< 0.029	10.47	271.2-252.9	n/a	7.3E-02	1.9E+01
estrone (E1)	< 0.00073	< 0.00073	15.50	504.1-171.1	504.1-156.0	7.3E-05	3.8E+00
estradiol (E2)	< 0.00073	< 0.00037	15.64	506.0-171.2	506.0-128.0	5.5E-04	4.5E+00
testosterone (T)	4.9 ± 5.3 *	2.3 ± 2.4 *	9.59	289.0-108.7	289.0-97.1	3.7E-03	2.3E+01
dihydrotestosterone (DHT)	0.15 ± 0.22	0.098 ± 0.12	11.92	297.1-255.0	291.1-91.2	3.7E-03	2.3E+01
3?- and 3?-diols (DIOLS)	34 ± 27 ^†^	<7.5	11.92	275.3-257.3	n/a	7.5E+00	3.0E+01

Serum steroid concentrations are reported as ng/mL. Bold indicates statistical significance by t-test. * indicates a significant decrease in variance. † indicates that diols are outside of standard curve.

## DISCUSSION

One of the prevailing views is that wild-type mice do not spontaneously develop prostate disease; this could be due to several reasons including: the lack of anatomical description, as well as, functional urinary events and their associations [37,38]. In spontaneously hypertensive rats, prostatic hyperplasia and associated urinary dysfunction occurs with age [39]. Our studies show that aged mice have similar changes within the lower urinary tract corresponding to changes seen in the hormone-induced mouse model of LUTD. This suggests that as mice age, there is a development of prostate related disease that corresponds to human disease. Although the anatomical differences of the prostate lobe make prostate changes in the lobes of the mouse difficult to directly correlate with prostate changes within the human, the anatomy of the prostatic urethra is more consistent with that of the human. The prostatic urethra is the region entirely enclosed by a “capsule”, the muscular rhabdosphincter, where the prostatic ducts, ejaculatory ducts, and vas deferens insert and run in parallel before opening into the post-prostatic urethra. Because this region is enclosed by skeletal muscle, any increases in prostatic duct number, cells expressing SMA, or extracellular matrix could impinge upon the urethral lumen, further decreasing the diameter at this narrowest urethral section.

One factor of BPH development in human disease is an increase in prostate proliferation, leading to the development of nodules. Our examination of mouse prostate proliferation rate using BrdU incorporation shows that aging does not change the rate by which normal prostate renews. This is consistent with previously published data showing that after puberty, the prostate is quiescent with ~1% overall proliferation. Our study is unique in that it examines proliferation over a 4-week window with BrdU accumulation, as opposed to the accumulation of proliferation over two years. Under normal conditions, there is < 0.5% apoptosis within the prostate lobes and our TUNEL staining shows the same percentage of apoptosis within the prostate in both young and aged mice [40]. While our examination of apoptosis at one time point cannot be directly compared to the incorporation of BrdU over the course of a week, the increase in prostate duct number within the prostatic urethra suggests an accumulation of prostatic proliferation over the lifespan of the mouse consistent with prostate enlargement, i.e. there is an increase in prostate lobe mass with aging. However, this increased proliferation within the prostate lobes and prostatic urethra in mice does not lead to the formation of nodules. Collectively, the increase in non-nodular prostatic tissue around the prostatic urethra could impinge and decrease the urethral lumen resulting in LUTD.

The deposition of collagen within the prostate and around the urethra has also been shown to be a factor in human disease as well as playing a role in BPH treatment failure [31,41,42]. Our studies show a significant correlation of age and collagen deposition and remodeling. Not only is there an increase in total collagen due to age, age also significantly increases the medium/large collagen bundles consistent with active fibrosis. This accumulation, specifically within the prostatic urethra, could significantly alter voiding by decreasing urethral compliance thus interfering with maximal dilation of the urethra during urination. Our examination of changes in smooth muscle showed no age-related increase or decrease of SMA positive cells within the prostate lobes or prostatic urethra. However, we have not examined changes in muscle contractility or innervation that could affect urethral lumen size and hence LUTD; the effect of age on these factors remain to be examined.

In humans, BPH incidence increases as a consequence of aging and coincides with the natural decrease in circulating T while E2 remains constant. The examination of androgens (T, DHT) by LC-MS^2^ show an age-related decrease that is similar to that of aging men. Additionally, the larger range of T concentration in young mice is significantly decreased with age. This suggests that steroid hormone changes within the mice are a risk factor for the development of prostate disease similar to that of humans.

All of the cellular changes within the prostate lobes and urethra consistent with human BPH also result in urinary dysfunction similar to LUTS experienced by patients. There is a significant increase in the number of voids, specifically of smaller diameter voids, with aging mice that are characteristic of LUTS (e.g. frequency, dribbling). Examination of flow rate through the entire lower urinary tract by ultrasound reveals the prostatic urethra as the narrowest region of the urethra, corresponding to the highest flow velocity. This significant change in velocity between the bladder neck, prostatic urethra, and post-prostatic urethra is lost with aging. Upon further examination of the urethra with MRI, age significantly increases the length of the prostatic urethra but not the diameter. This lengthening with age could increase urethral resistance (*R* = 8*Lɳ*/(*π*·*r*^4^), where L is length) and hence serve as an obstruction potentially leading to bladder compensation.

**Figure 8:**
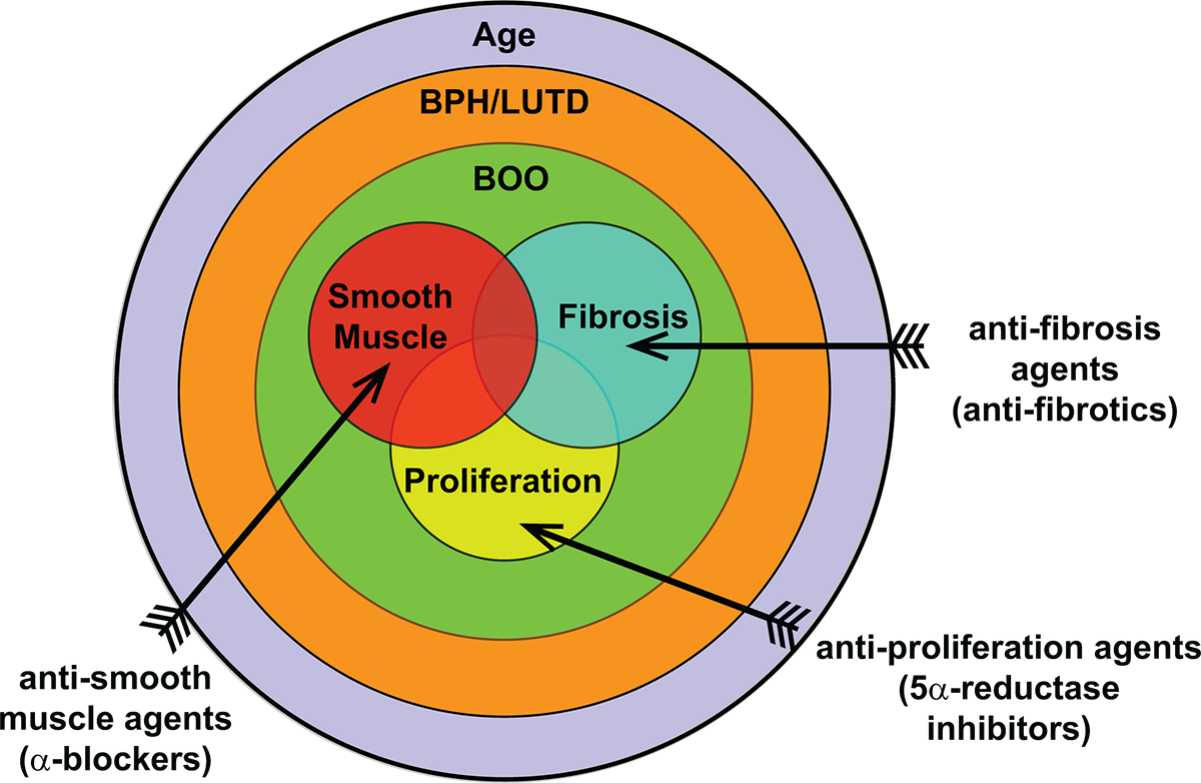
**Overall contribution of age to BPH pathogenic mechanisms. BPH/LUTD is a disease that occurs through the normal course of aging and often manifests clinically as bladder outlet obstruction (BOO).** The main contributing factors of BOO include proliferation, smooth muscle, and fibrosis. Treatments currently include anti-proliferation agents (5ARI) and anti-smooth muscle agents (α-blockers); anti-fibrosis agents remain to be examined.

Our study, examining the lower urinary tract of aged mice, shows that age has a significant impact on urinary function compared to sexually mature young mice. Taken together, age impacts several contributing factors generally associated with BPH initiation and progression in humans (Figure 8). The use of younger mice in various models studying BPH has been invaluable and can recapitulate different aspects and symptoms of the disease. However, since age is significantly altering normal prostate and voiding function in mice and BPH is a disease of aging, the need to examine the contribution of age to the disease process needs to be examined. This could provide further insight on disease development and progression as well as treatment efficacy and resistance.

## MATERIALS AND METHODS

### Animals

All animal experiments were conducted under the protocols approved by the University of Wisconsin Animal Care and Use Committee. Male C57Bl6/J mice were obtained from The Jackson Laboratory (Bar Harbor, ME) or directly from the National Institute of Aging (Bethesda, MD). Animals were house under standard laboratory conditions with 12:12 light/dark cycle and provided with food and water *ad libitum*. Young mice were obtained at 8 weeks of age and aged mice were obtained at 24 months of age. Mice were euthanized with carbon dioxide followed by cardiac puncture or cervical dislocation following ultrasound. Three caliper measurements (x, y, z) were taken of each bladder and volume calculated by 4/3·π·((x·y·z)/8). Urogenital tracts were carefully dissected, and mass of tissue determined as previously described [17]. The bladder and urethra remained attached after dissection and were fixed in 10% normal buffered formalin (NBF) for imaging. Tissues for immunohistochemistry were fixed in NBF and paraffin embedded.

### Void spot assays

Void spot assays were performed as previously described [43]. Briefly, mice were placed individually on 16 x 26-cm thick chromatography paper (Ahlstrom, Kaukauna, WI) secured to standard mouse cages. Mice were on restricted from water during the 4 hour study duration. Filter papers were imaged with a BioRad ChemiDoc Imaging System under ultraviolet light using an ethidium bromide filter set and 0.5 second exposure (BioRad, Hercules, CA). Images were imported into ImageJ and void spots analyzed with VoidWhizzard [44].

### Ultrasound

Contrast-enhanced doppler ultrasound was performed on young and aged mice. After anaesthetizing the mice with isoflurane, bladders were catheterized via abdominal insertion. Once the bladder was visualized by ultrasound, the contrast agent, DEFINITY^®^ (Lantheus, Billerica, MA), was injected into the bladder through the catheter. Using Doppler, the contrast agent velocity was measured through the urethra at the bladder neck, prostatic urethra, and post-prostatic urethra. B-mode imaging was also used to measure the diameter at those respective points.

### Magnetic resonance imaging

Intact bladder and urethra were dissected from young and aged mice, agar embedded, and prepared for magnetic resonance imaging (MRI). MR imaging was performed in a 9.4T animal MRI scanner (Bruker, Billerica, MA) using a high-resolution 3D rapid acquisition with resolution enhancements (RARE) technique including inversion recovery to approximately null the signal from agar. The imaging parameters were: repetition time (TR): 4000 ms, inversion time (TI): 1346 ms, echo time (TE): 55.4 ms, echo train length (ETL): 16, pixel bandwidth: 312.5 Hz, acquisition matrix: 320x320x280, spatial resolution: 0.1x0.1x0.1 mm^3^.

### Measurement and reconstruction of selected endpoints

The MR images, containing data from the entire urogenital tract of 9 mice, were imported into MIMICS (Materialise, Leuven, Belgium) and 3-matic (Materialize). Key features were measured, including bladder volume, detrusor muscle volume, bladder neck diameter/volume/length, urethra diameters, urethra total length, and urethra segmental lengths (Figure 3, Table 2). The anatomical boundaries of each urogenital tract were identified and manually segmented in MIMICS (Figure 3). Once each boundary was delineated, 3D models of each urogenital tract were processed in 3-matic. Bladder lumen and detrusor muscle volumes were calculated by delineating the 3D bladder fluid volume from the bladder wall and then calculating the volume of each by totaling the number of voxels in each part. Bladder neck diameter was determined by finding the mean tangent points between the urethra opening and the bladder wall on the opposing sides of the bladder (Figure 3). Urethral diameters, total length, and segmental lengths were found by segmenting and measuring the urethral opening at various points on the 3D segmented model.

### BrdU incorporation

To examine proliferation by age and across time by BrdU incorporation, young (8 week) and aged (24 month) mice were administered 1mg/mL BrdU in water for 5 days [45]. Drinking water consumption was monitored and was unaffected by BrdU. Following 5 days of BrdU, the mice were returned to normal water for 3 weeks.

### Immunohistochemistry

All procedures were performed as previously described [17]. Briefly, 5μm sections were cleared and rehydrated. Antigen retrieval was performed using a Decloaking Chamber^TM^ (Biocare Medical, Pacheco, CA) in 10mM citrate buffer for 15 minutes at 100^0^C. Sections were stained with anti-BrdU (1:200) to assess proliferation. Images were captured using Nuance (Perkin-Elmer, Waltham, MA) and quantified using InForm software (Perkin-Elmer). For the examination of smooth muscle, sections were stained with anti-smooth muscle α-actin (1:800). Images were captured using a Nikon epifluorescence microscope. Percent of smooth muscle α-actin was quantified using ImageJ by examining the percentage of positivity to total tissue, excluding any luminal space.

### Evaluation of collagen

The midpoint of the prostatic urethra was identified from serial sections as previously described [20]. To identify collagen bundle sizes, picrosirius red staining was performed according to manufacturer’s protocol. Images were captured under circular polarized light to capture collagen fibers in every orientation. Collagen bundle size was quantified using ImageJ as previously described [46].

### Serum steroid extraction and derivatization

A two-stage liquid-liquid extraction of steroids from mouse serum was performed. Mouse serum samples (300 µL) and calibration curve standards were transferred to 5 mL glass test tubes for extraction. Ultrapure HPLC-grade bottled water (500 µL, Fisher Scientific, Hampton, NH) and stable isotope-labeled internal standards were added to each as follows: 200 pg each d9-progesterone and d5-testosterone, and 50 pg d5-estradiol. Methyl tert-butyl ether (2 mL, Fisher Scientific) was added, followed by vigorous vortexing for 5 min and centrifugation at 530 × *g* (1,500 rpm) for 3 min at 4^0^C. The organic phase (top layer) containing steroid hormones was transferred to a new glass test tube via glass pipette and evaporated to dryness via air stream in a heated water bath at 60^0^C. Dried samples were dissolved in ethanol (200 µL) and diluted in ultrapure water (500 µL). Dichloromethane (1 mL, Fisher Scientific) was added for the second liquid-liquid extraction, followed by vortexing, centrifugation, organic transfer (bottom layer), and dry-down as above. To derivatize estrogens (E1 and E2), dried samples were dissolved in 25 µL of 0.1 M NaHCO_3_ buffer followed by the addition of 5 mg dansyl chloride in acetonitrile (at 200 mg/mL) and incubation at 40^0^C for 4 min. This reaction mixture (50 µL) was transferred to TruView minivials (Waters Acquity, Milford, MA) and stored at 4^0^C until liquid chromatography-tandem mass spectrometry (LC-MS^2^) analysis.

### Liquid chromatography-tandem mass spectrometry analysis

The LC-MS^2^ analysis of the extracted mouse serum samples was performed with an ultrahigh performance liquid chromatography (UPLC) system (Waters Acquity) interfaced to a hybrid triple quadrupole-linear ion trap mass spectrometer using an atmospheric pressure chemical ionization source (AB Sciex QTRAP 5500, Framingham, MA). Waters Acquity UPLC Console v1.50 software was used to control the UPLC and Analyst v1.6 software (Sciex, Concord, ON) was used to control the mass spectrometer. Samples, calibration curve standards, and quality control samples were ordered randomly, and blanks were analyzed between each. Samples (10 µL) were separated in the reverse phase via C18 column (2.1 x 100 mm, 2.6 µm, 100Å; Phenomenex Kinetex, Torrence, CA) using water with 0.1% formic acid (mobile phase A) and methanol with 0.1% formic acid (mobile phase B), at 250 µL/min (Optima solvents, Fisher Scientific). Column temperature was 35^0^C and samples were kept at 10^0^C throughout the experiment. The LC gradient was as follows: 0-1 minute 45% B, 1-12.5 min 45-67.5% B, 12.5-13 min 67.5-85% B, 13-14 min 85-87% B, 14-14.5 min 87-92% B, 14.5-17 min 92-96% B, 17-17.5 min 96-98% B, 17.5-18 min 98% B, 18-18.5 min 98-45% B, 18.5-20 min 45% B. The mass spectrometer was operated in positive-ion mode with the following settings: curtain gas, 30 psi; corona discharge current, 3 V; collision-induced dissociation gas, medium; nebulizing gas, 25 psi; entrance potential, 10 V; and source temperature, 500^0^C. Internal standards were d9-progesterone for progesterone, d5-E2 for E1 and E2, and d5-testosterone for all other analytes. Data were processed via Analyst v1.5.1 and MultiQuant software (Sciex). Calibration curves (≥5 points) were acceptable when all points fell within 20% of the response function (1/x) and r>0.99. Quantitative results were calculated using this calibration curve and the response factor of each analytes’ multiple reaction monitoring (MRM) area counts relative to that of its internal standard.

### Statistics

Statistical significance for lower urinary tract morphometrics, proliferation, and collagen deposition was calculated using Student’s t-test. Statistical significance for LC-MS^2^ was examined with Student’s t-test and F-test. Statistical significance for the urethral diameter and voiding velocity was examined with one-way and two-way ANOVA.

## References

[1] Rullis I, Shaeffer JA, Lilien OM. Incidence of prostatic carcinoma in the elderly. Urology. 1975; 6:295–97. 10.1016/0090-4295(75)90749-9 1172317

[2] Sakr WA, Grignon DJ, Crissman JD, Heilbrun LK, Cassin BJ, Pontes JJ, Haas GP. High grade prostatic intraepithelial neoplasia (HGPIN) and prostatic adenocarcinoma between the ages of 20-69: an autopsy study of 249 cases. In Vivo. 1994; 8:439–43. 7803731

[3] Berry SJ, Coffey DS, Walsh PC, Ewing LL. The development of human benign prostatic hyperplasia with age. J Urol. 1984; 132:474–79. 10.1016/S0022-5347(17)49698-4 6206240

[4] Sakr WA, Haas GP, Cassin BF, Pontes JE, Crissman JD. The frequency of carcinoma and intraepithelial neoplasia of the prostate in young male patients. J Urol. 1993; 150:379–85. 10.1016/S0022-5347(17)35487-3 8326560

[5] Kirby RS. The natural history of benign prostatic hyperplasia: what have we learned in the last decade? Urology. 2000 (Suppl 1); 56:3–6. 10.1016/S0090-4295(00)00747-0 11074195

[6] Kortt MA, Bootman JL. The economics of benign prostatic hyperplasia treatment: a literature review. Clin Ther. 1996; 18:1227–41. 10.1016/S0149-2918(96)80078-6 9001839

[7] Isaacs JT, Coffey DS. Etiology and disease process of benign prostatic hyperplasia. Prostate Suppl. 1989; 2:33–50. 10.1002/pros.2990150506 2482772

[8] Chute CG, Panser LA, Girman CJ, Oesterling JE, Guess HA, Jacobsen SJ, Lieber MM. The prevalence of prostatism: a population-based survey of urinary symptoms. J Urol. 1993; 150:85–89. 10.1016/S0022-5347(17)35405-8 7685427

[9] Harman SM, Metter EJ, Tobin JD, Pearson J, Blackman MR, and Baltimore Longitudinal Study of Aging. Longitudinal effects of aging on serum total and free testosterone levels in healthy men. J Clin Endocrinol Metab. 2001; 86:724–31. 10.1210/jcem.86.2.7219 11158037

[10] Bjørnerem A, Straume B, Midtby M, Fønnebø V, Sundsfjord J, Svartberg J, Acharya G, Oian P, Berntsen GK. Endogenous sex hormones in relation to age, sex, lifestyle factors, and chronic diseases in a general population: the Tromsø Study. J Clin Endocrinol Metab. 2004; 89:6039–47. 10.1210/jc.2004-0735 15579756

[11] Bélanger A, Candas B, Dupont A, Cusan L, Diamond P, Gomez JL, Labrie F. Changes in serum concentrations of conjugated and unconjugated steroids in 40- to 80-year-old men. J Clin Endocrinol Metab. 1994; 79:1086–90. 7962278 10.1210/jcem.79.4.7962278

[12] Carter HB, Pearson JD, Metter EJ, Chan DW, Andres R, Fozard JL, Rosner W, Walsh PC. Longitudinal evaluation of serum androgen levels in men with and without prostate cancer. Prostate. 1995; 27:25–31. 10.1002/pros.2990270106 7541528

[13] Pierorazio PM, Ferrucci L, Kettermann A, Longo DL, Metter EJ, Carter HB. Serum testosterone is associated with aggressive prostate cancer in older men: results from the Baltimore Longitudinal Study of Aging. BJU Int. 2010; 105:824–29. 10.1111/j.1464-410X.2009.08853.x 19751256 PMC2848292

[14] Trifiro MD, Parsons JK, Palazzi-Churas K, Bergstrom J, Lakin C, Barrett-Connor E. Serum sex hormones and the 20-year risk of lower urinary tract symptoms in community-dwelling older men. BJU Int. 2010; 105:1554–59. 10.1111/j.1464-410X.2009.09090.x 20002438

[15] Ricke WA, Ishii K, Ricke EA, Simko J, Wang Y, Hayward SW, Cunha GR. Steroid hormones stimulate human prostate cancer progression and metastasis. Int J Cancer. 2006; 118:2123–31. 10.1002/ijc.21614 16331600

[16] Ricke WA, McPherson SJ, Bianco JJ, Cunha GR, Wang Y, Risbridger GP. Prostatic hormonal carcinogenesis is mediated by in situ estrogen production and estrogen receptor alpha signaling. FASEB J. 2008; 22:1512–20. 10.1096/fj.07-9526com 18055862

[17] Nicholson TM, Ricke EA, Marker PC, Miano JM, Mayer RD, Timms BG, vom Saal FS, Wood RW, Ricke WA. Testosterone and 17β-estradiol induce glandular prostatic growth, bladder outlet obstruction, and voiding dysfunction in male mice. Endocrinology. 2012; 153:5556–65. 10.1210/en.2012-1522 22948219 PMC3473198

[18] Flurkey K, Currer JM, Harrison DE. (2007). Mouse Models in Aging Research. In: Fox JG, Barthold SW, Davisson MT, Newcomer CE, Quimby FW, Smith AL, eds. The Mouse in Biomedical Research, 2nd Edition. New York; Elsevier, 2007, Volume 3:637-72.

[19] Bianchi-Frias D, Vakar-Lopez F, Coleman IM, Plymate SR, Reed MJ, Nelson PS. The effects of aging on the molecular and cellular composition of the prostate microenvironment. PLoS One. 2010; 5:e12501. 10.1371/journal.pone.0012501 20824135 PMC2931699

[20] Ricke WA, Timms BG, Vom Saal FS. (2018). Prostate Structure. In: Skinner M, ed. Encyclopedia of Reproduction: Academic Press.

[21] Franks LM, Payne J. The influence of age on reproductive capacity in C57BL mice. J Reprod Fertil. 1970; 21:563–65. 5442326 10.1530/jrf.0.0210563

[22] Turturro A, Witt WW, Lewis S, Hass BS, Lipman RD, Hart RW. Growth curves and survival characteristics of the animals used in the Biomarkers of Aging Program. J Gerontol A Biol Sci Med Sci. 1999; 54:B492–501. 10.1093/gerona/54.11.B492 10619312

[23] Liu TT, Rodgers AC, Nicholson TM, Macoska JA, Marker PC, Vezina CM, Bjorling DE, Roldan-Alzate A, Hernando D, Lloyd GL, Hacker TA, Ricke WA. Ultrasonography of the adult male urinary tract for urinary functional testing. Jove. 2019. https://www.jove.com/video/59802/ultrasonography-adult-male-urinary-tract-for-urinary-functional 10.3791/59802 PMC7328372 31475976

[24] Cunha GR, Vezina CM, Isaacson D, Ricke WA, Timms BG, Cao M, Franco O, Baskin LS. Development of the human prostate. Differentiation. 2018; 103:24–45. 10.1016/j.diff.2018.08.005 30224091 PMC6234090

[25] Phillips TR, Wright DK, Gradie PE, Johnston LA, Pask AJ. A Comprehensive Atlas of the Adult Mouse Penis. Sex Dev. 2015; 9:162–72. 10.1159/000431010 26112156 PMC5012965

[26] Timms BG. Prostate development: a historical perspective. Differentiation. 2008; 76:565–77. 10.1111/j.1432-0436.2008.00278.x 18462432

[27] Timms BG, Mohs TJ, Didio LJ. Ductal budding and branching patterns in the developing prostate. J Urol. 1994; 151:1427–32. 10.1016/S0022-5347(17)35273-4 8158800

[28] Hollabaugh RS Jr, Dmochowski RR, Steiner MS. Neuroanatomy of the male rhabdosphincter. Urology. 1997; 49:426–34. 10.1016/S0090-4295(96)00497-9 9123709

[29] Gao S, Wu H, Wang F, Wang Z. Altered differentiation and proliferation of prostate epithelium in mice lacking the androgen receptor cofactor p44/WDR77. Endocrinology. 2010; 151:3941–53. 10.1210/en.2009-1080 20519372 PMC2940529

[30] Wu CT, Altuwaijri S, Ricke WA, Huang SP, Yeh S, Zhang C, Niu Y, Tsai MY, Chang C. Increased prostate cell proliferation and loss of cell differentiation in mice lacking prostate epithelial androgen receptor. Proc Natl Acad Sci USA. 2007; 104:12679–84. 10.1073/pnas.0704940104 17652515 PMC1937526

[31] Bauman TM, Nicholson TM, Abler LL, Eliceiri KW, Huang W, Vezina CM, Ricke WA. Characterization of fibrillar collagens and extracellular matrix of glandular benign prostatic hyperplasia nodules. PLoS One. 2014; 9:e109102. 10.1371/journal.pone.0109102 25275645 PMC4183548

[32] Bell-Cohn A, Mazur DJ, Hall C, Schaeffer AJ, Thumbikat P. Uropathogenic Escherichia coli-induced fibrosis, leading to lower urinary tract symptoms, is associated with type 2 cytokine signaling. Am J Physiol Renal Physiol. 2019; 316:F682–92. 30623726 10.1152/ajprenal.00222.2018 PMC6483034

[33] Macoska JA, Wang Z, Virta J, Zacharias N, Bjorling DE. Inhibition of the CXCL12/CXCR4 axis prevents periurethral collagen accumulation and lower urinary tract dysfunction in vivo. Prostate. 2019; 79:757–67. 10.1002/pros.23781 30811623 PMC7269149

[34] Nicholson TM, Moses MA, Uchtmann KS, Keil KP, Bjorling DE, Vezina CM, Wood RW, Ricke WA. Estrogen receptor-α is a key mediator and therapeutic target for bladder complications of benign prostatic hyperplasia. J Urol. 2015; 193:722–29. 10.1016/j.juro.2014.08.093 25167991 PMC4305478

[35] Wu WF, Maneix L, Insunza J, Nalvarte I, Antonson P, Kere J, Yu NY, Tohonen V, Katayama S, Einarsdottir E, Krjutskov K, Dai YB, Huang B, et al. Estrogen receptor β, a regulator of androgen receptor signaling in the mouse ventral prostate. Proc Natl Acad Sci USA. 2017; 114:E3816–22. 10.1073/pnas.1702211114 28439009 PMC5441728

[36] Machida T, Yonezawa Y, Noumura T. Age-associated changes in plasma testosterone levels in male mice and their relation to social dominance or subordinance. Horm Behav. 1981; 15:238–45. 10.1016/0018-506X(81)90013-1 7298026

[37] Li J, Tian Y, Guo S, Gu H, Yuan Q, Xie X. Testosterone-induced benign prostatic hyperplasia rat and dog as facile models to assess drugs targeting lower urinary tract symptoms. PLoS One. 2018; 13:e0191469. 10.1371/journal.pone.0191469 29351556 PMC5774778

[38] Suwa T, Nyska A, Haseman JK, Mahler JF, Maronpot RR. Spontaneous lesions in control B6C3F1 mice and recommended sectioning of male accessory sex organs. Toxicol Pathol. 2002; 30:228–34. 10.1080/019262302753559560 11950166

[39] Matityahou A, Rosenzweig N, Golomb E. Rapid proliferation of prostatic epithelial cells in spontaneously hypertensive rats: a model of spontaneous hypertension and prostate hyperplasia. J Androl. 2003; 24:263–69. 10.1002/j.1939-4640.2003.tb02671.x 12634314

[40] Ma X, Ziel-van der Made AC, Autar B, van der Korput HA, Vermeij M, van Duijn P, Cleutjens KB, de Krijger R, Krimpenfort P, Berns A, van der Kwast TH, Trapman J. Targeted biallelic inactivation of Pten in the mouse prostate leads to prostate cancer accompanied by increased epithelial cell proliferation but not by reduced apoptosis. Cancer Res. 2005; 65:5730–39. 10.1158/0008-5472.CAN-04-4519 15994948

[41] Ma J, Gharaee-Kermani M, Kunju L, Hollingsworth JM, Adler J, Arruda EM, Macoska JA. Prostatic fibrosis is associated with lower urinary tract symptoms. J Urol. 2012; 188:1375–81. 10.1016/j.juro.2012.06.007 22906651 PMC3485634

[42] Hao L, Greer T, Page D, Shi Y, Vezina CM, Macoska JA, Marker PC, Bjorling DE, Bushman W, Ricke WA, Li L. In-depth characterization and validation of human urine metabolomes reveal novel metabolic signatures of lower urinary tract symptoms. Sci Rep. 2016; 6:30869. 10.1038/srep30869 27502322 PMC4977550

[43] Keil KP, Abler LL, Altmann HM, Bushman W, Marker PC, Li L, Ricke WA, Bjorling DE, Vezina CM. Influence of animal husbandry practices on void spot assay outcomes in C57BL/6J male mice. Neurourol Urodyn. 2016; 35:192–98. 10.1002/nau.22692 25394276 PMC4428995

[44] Wegner KA, Abler LL, Oakes SR, Mehta GS, Ritter KE, Hill WG, Zwaans BM, Lamb LE, Wang Z, Bjorling DE, Ricke WA, Macoska J, Marker PC, et al. Void spot assay procedural optimization and software for rapid and objective quantification of rodent voiding function, including overlapping urine spots. Am J Physiol Renal Physiol. 2018; 315:F1067–80. 29972322 10.1152/ajprenal.00245.2018 PMC6230749

[45] Sangiorgi E, Capecchi MR. Bmi1 lineage tracing identifies a self-renewing pancreatic acinar cell subpopulation capable of maintaining pancreatic organ homeostasis. Proc Natl Acad Sci USA. 2009; 106:7101–06. 10.1073/pnas.0902508106 19372370 PMC2678421

[46] Whittaker P, Kloner RA, Boughner DR, Pickering JG. Quantitative assessment of myocardial collagen with picrosirius red staining and circularly polarized light. Basic Res Cardiol. 1994; 89:397–410. 10.1007/BF00788278 7535519

